# Life cycle phases: Literature review and new classification proposal for application in healthcare

**DOI:** 10.1590/2177-6709.28.5.e23spe5

**Published:** 2023-11-10

**Authors:** Luciane Macedo de MENEZES, Bruno Moreira das NEVES, Bruna Barnard MOTTA, Fabiane AZEREDO, Cátia Cardoso Abdo QUINTÃO

**Affiliations:** 1 Pontifical Catholic University of Rio Grande do Sul, School of Health and Life Sciences (Porto Alegre/RS, Brazil).; 2 State University of Rio de Janeiro, School of Dentistry, Department of Preventive and Community Dentistry (Rio de Janeiro/RJ, Brazil).; 3 Brazilian Society of Dental Surgeons - RS (SOBRACID/IMED) (Porto Alegre/RS, Brazil).

**Keywords:** Life cycle, Life cycle stages, Age group, Orthodontics

## Abstract

**Introduction:**

Although uncommon in dentistry, the concept of the life cycle holds great importance for dental professionals in identifying crucial intervention opportunities and determining the optimal timing for treatments and procedures.

**Objective:**

To carry out a review of the literature on life cycle classifications and their distinct phases, evaluating their applicability in healthcare.

**Methods:**

A literature review was performed, searching for articles in PubMed, SciELO, National Health Library (BvB), and Google Scholar databases, as well as relevant books. The keywords “life cycle,” “life stages,” “human development,” “age groups,” and “biological age” were used. Relevant articles were selected by analyzing their titles and abstracts, and read in full to confirm their inclusion in the research.

**Results:**

Nine distinct life cycle classifications were found, each with unique criteria.

**Conclusion:**

Based on the comprehensive literature review, a novel classification was proposed (The 10-phase Life Cycle), which encompasses dental, growth, physiological aging, sociocultural, and behavioral characteristics, aiming to enhance communication among healthcare professionals, particularly those engaged in the growth, development, and aging processes of human beings.

## RESUMO

**Introdução:**

Embora incomum na Odontologia, o conceito de ciclo vital é de grande importância para ajudar os profissionais dessa área a identificar oportunidades cruciais para intervenções e para determinar o momento ideal para tratamentos e procedimentos.

**Objetivo:**

Realizar uma revisão da literatura sobre as classificações do ciclo vital e suas distintas fases, avaliando sua aplicabilidade na área da saúde.

**Métodos:**

Foi realizada uma revisão da literatura, buscando artigos nas bases de dados PubMed, SciELO, Biblioteca Nacional de Saúde (BvB) e Google Acadêmico; além de livros relevantes. Foram utilizadas as palavras-chave “ciclo vital”, “estágios de vida”, “desenvolvimento humano”, “faixas etárias” e “idade biológica”. Os artigos relevantes foram selecionados por meio da análise de seus títulos e resumos e lidos na íntegra para confirmar sua inclusão na pesquisa.

**Resultados:**

Nove classificações distintas de ciclo vital foram encontradas, cada uma com critérios únicos.

**Conclusão:**

Com base nessa revisão abrangente da literatura, é proposta uma nova classificação (o ciclo vital de 10 fases) que engloba características dentárias, de crescimento, envelhecimento fisiológico, socioculturais e comportamentais, com o objetivo de melhorar a comunicação entre os profissionais da área de saúde, particularmente aqueles envolvidos nos processos de crescimento, desenvolvimento e envelhecimento do ser humano.

## INTRODUCTION

The increase in life expectancy has led to increased longitudinal studies and studies related to the life course, including the changes that occur during the life cycle.

A person’s life cycle is commonly divided into sequential phases, which follow their age, from birth to death. These phases, which have a predictable duration, can also be demarcated from a perspective of biological events (puberty, menarche, reproduction, and menopause) and from a social events perspective (marriage, parenting, and retirement).^1^ Thus, the life cycle phases can be measured from developmental markers, chronologically defined, considering the amount of time elapsed from conception or birth to the end of life.^2^ Chronological age can be a reference axis for persons, and it has the advantage of presenting similar conditions according to sex, race, or social status.^3^ However, because of social, historical, and cultural changes, the delimitation of life phases has been changing, making the chronological criterion valid but insufficient to characterize the life cycle,^4^ since persons of the same age may present different biological conditions.^3^ In Western societies, age groups chronologically organized, based on a chronological system, are a crucial tool even for defining and attributing status -such as legal adulthood status-, insertion in the labor market and the right to retirement.^5^ However, the use of chronological age, without detailing the criteria used,^6^ requires more complete classifications.

The use of well-defined phases of the life cycle in the health area is essential for professionals to recognize the windows of opportunity for intervention and the most appropriate time to conduct treatments. Identifying the characteristics of each phase of the life cycle becomes essential for health professionals to act appropriately in growth, development, and aging. This knowledge makes it possible to understand the normal patterns, conflicts, and expected disorders,^7^ besides facilitating communication between professionals. However, establishing a standard for human life is a challenge because this reflects several aspects, including cultural and social ones.^8^

The present study comprehensively reviewed the existing literature on life cycle classifications, considering their distinct phases. Based on the findings, a novel life cycle classification was proposed, based on dental, growth, physiological aging, sociocultural, and behavioral characteristics that facilitate communication between health professionals, particularly those who act in human growth, development, and aging.

## METHODS

**Figure 1: f1:**
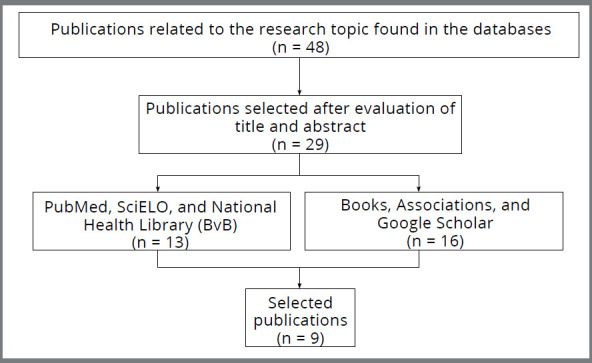
Flowchart of collection of bibliographic material to carry out the research.

A review of the literature was carried out, searching for articles in the PubMed, SciELO, National Health Library (BvB), and Google Scholar databases, from 1972 to 2020. The Portuguese keywords “*ciclo vital*”, “*estágios do ciclo de vida*”, “*desenvolvimento humano*”, “*faixas etárias*”, “*grupos etários*”, “*idade biológica*”, and their equivalents in English (“*life cycle*,” “*life cycle stages*”, “*human development*”, “*age groups*”) were used. The terms were associated with each other by the Portuguese operators “E” and “OU,” and their English counterparts, “AND” and “OR.” From the search results, the pertinent publications were selected through the analysis of their titles and abstracts, both in Portuguese and in English. The chosen articles were thoroughly read to confirm whether they should be included or excluded from the research. The criteria for inclusion encompassed articles and book chapters that were published with full text, in Portuguese or English, presenting characterizations of the life cycle phases, with no limitation on publication date (Fig 1). In order to identify other relevant publications, the reference list of the included studies was manually checked for relevant or missing studies using the keywords previously mentioned. Studies that met all inclusion criteria were selected. When there was no mutual agreement between the two authors responsible for selecting the articles, a third author was involved to make a final decision. The literature review was divided according to the different classifications found, and the results were presented and discussed.

[Fig f1]

## RESULTS

Based on the bibliographic material consulted, 9 classifications were registered for the life cycle, which will be described according to the number of phases, presenting their criteria and nomenclatures. From these nine classifications found, graphs with colors were created, representing the life cycle phases, as described by each author.

### LIFE CYCLE CLASSIFICATIONS:

#### THE 3-PHASE LIFE CYCLE (CAMARANO, 2006)

**Figure 2:**
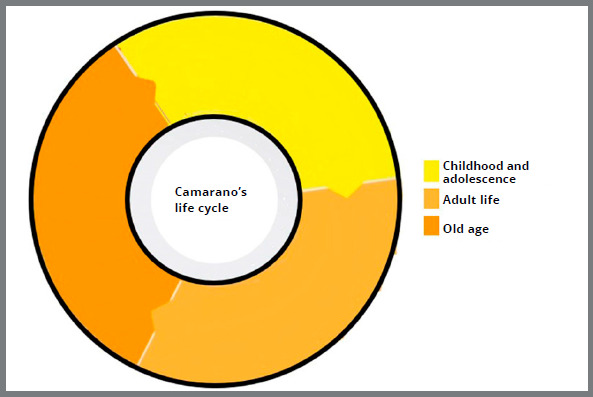
Graph with life cycle in 3 phases, based on Camarano’s classification (2006).^1^

According to Camarano,^1^ the life cycle is divided into three phases, the first being a junction between childhood and adolescence, the second represented by adulthood, and the third corresponding to old age (Fig 2). The life cycle organized in this configuration concerns the person’s position in the labor market and the family constitution.^1^

#### THE 5-PHASE LIFE CYCLE (BOGIN & SMITH, 1996)

**Figure 3:**
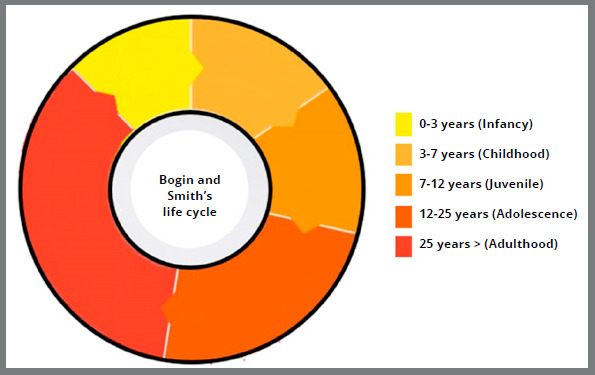
Graph with life cycle in 5 phases, based on Bogin and Smith’s classification (1996).^9^

According to Bogin and Smith,^9^ after birth, the life cycle is described in five phases: infancy, childhood, juvenile, adolescence, and adulthood (Fig 3). These phases are characterized as follows:

» Infancy: starts with birth, or, more accurately, when the person first receives nutrition via lactation, be it exclusive or non-exclusive breastfeeding, and ends with weaning. In pre-industrialized societies, this occurs from 0 to 36 months.
» Childhood: the period after weaning, in which the person still depends on someone for food that must be prepared considering the dentition and digestive tract immaturity, for protecting the person against diseases. The important milestones for the completion of the stage are the eruption of the first permanent molar and complete growth of the brain, which correspond to the possibility of nutrition like that of an adult, because of the masticatory capacity and increased cognitive capacity. The events that determine the end of childhood occur around 6.5 to 7 years of age.
» Juvenile: phase preceding puberty, in which persons are no longer so dependent on their parents for food^10^ and have greater physical and cognitive capacity.^11^ ^,^ ^12^ For girls, this period ends around the age of 10, while for boys it occurs 2 years later.
» Adolescence: starts with puberty, when signs of sexual maturation are manifested, such as the appearance of pubic hair. In this phase, growth is accelerated, characterizing the “growth spurt”. The end of this phase is related to the achievement of stature and complete sexual maturity. For women, it occurs, on average, at 19 years of age, and in men, around 21 to 25 years.
» Adulthood: bone apposition ends, and resorption begins around the fifth decade of life.

#### THE 6-PHASE LIFE CYCLE (HAVIGHURST, 1972)

**Figure 4:**
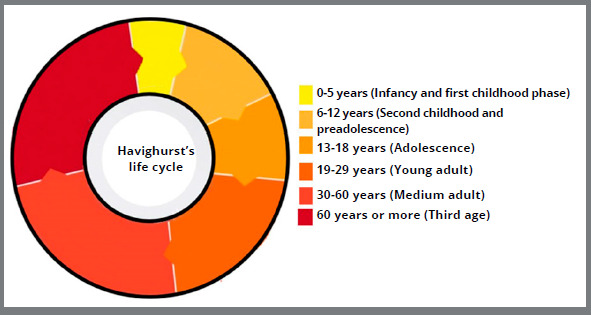
Graph with life cycle in 6 phases, based on Havighurst’s classification (1972).^13^

This classification of the life cycle into six phases (Fig 4) is based on developmental tasks, as proposed by Havighurst^13^:

» Infancy and first childhood phase: from birth to 5 years of age; period in which learning to walk, speak, eat solid foods, and readiness to read occur.
» Second childhood and preadolescence: from 6 to 12 years of age, when personal independence and appropriate sex role learning are achieved.
» Adolescence: from 13 to 18 years of age; phase in which mature relationships are maintained, and physical acceptance and career preparation occur.
» Young adult: from 19 to 29 years of age; period of choosing a partner, family formation, and civic responsibility.
» Medium adult: the phase between 30 and 60 years of age, when the career achieves more satisfactory results, adapting to the aging of the parents.
» Third age: 60 years or more, corresponds to the retirement phase, understanding of the proximity to the end-of-life, reduction of income, strength, and health.

#### THE 6-PHASE LIFE CYCLE (UNITED NATIONS, 1982)

**Figure 5:**
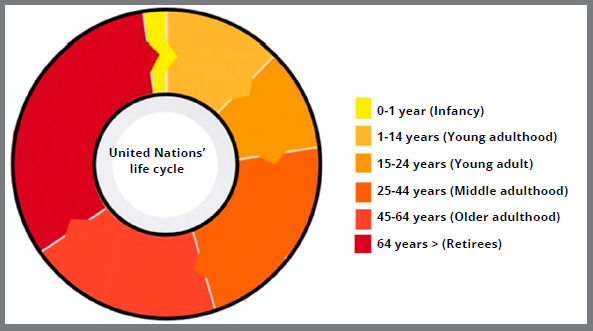
Graph with life cycle in 6 phases, based on the United Nations classification (1982).^14^

The United Nations^14^ proposed a classification in six phases (Fig 5):

» Infancy (under 1-year old).
» Youth (1 to 14 years).
» Young adulthood (15 to 24 years).
» Middle adulthood (25 to 44 years).
» Older adulthood (45 to 64 years).
» Retirement age (65 years and beyond).

The United Nations addresses different definitions, with a greater or lesser range of ages, for use according to the required purpose, subdividing them into the following phases: preschool children (from 1 to 4 years); school-age children (from 5 to 14 years); adolescence (phase between childhood and adulthood that comprises the ages from 10 to 19 years, with rapid physical, cognitive and psychosocial growth); third age (persons aged 64 years or older).

#### THE 7-PHASE LIFE CYCLE (GALLAHUE ET AL., 2013)

**Figure 6:**
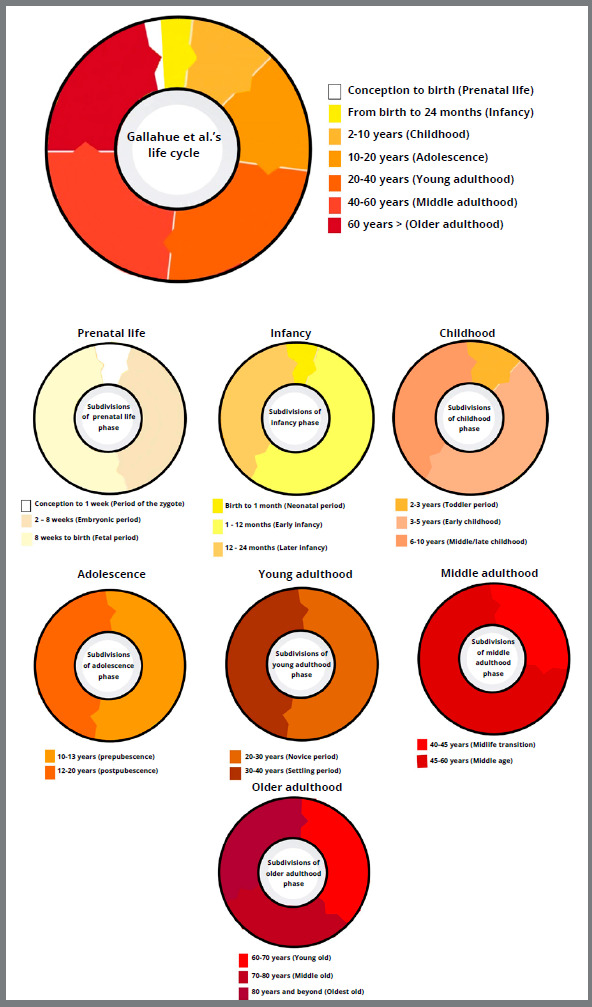
Graph with life cycle in 7 phases, based on the classification by Gallahue et al.^15^ (2013), with its subdivisions.

The classification of the life cycle into seven phases is based on chronological criteria with subdivisions proposed by Gallahue et al.^15^ (Fig 6):

» Prenatal life (conception to birth):
  - *Period of the zygote* - conception to 1 week;
  - *Embryonic period* - 2 to 8 weeks;
  - *Fetal period* - 8 weeks to birth.
» Infancy (from birth to 24 months):
  - *Neonatal period*
  - *Early infancy*
  - *Later infancy*
» Childhood (2 to 10 years):
  - *Toddler period* - 2 and 3 years;
  - *Early childhood* - 3 to 5 years;
  - *Middle/late childhood* - 6 to 10 years.
» Adolescence (10 to 20 years):
  - *Prepubescence* - 10 to 12 years for girls/11 to 13 years for boys;
  - *Postpubescence* - 12 to 18 years for girls/14 to 20 years for boys.
» Young adulthood (20 to 40 years):
  - *Novice period* - 20 to 30 years;
  - *Settling period* - 30 to 40 years.
» Middle adulthood (40 to 60 years):
  - *Midlife transition* - 40 to 45 years;
  - *Middle age* - 45 to 60 years.
» Older adulthood (over 60 years):
  - *Young old* - 60 to 70 years;
  - *Middle old* - 70 to 80 years;
  - *Oldest-old* - 80 years and beyond.

#### THE 8-PHASE LIFE CYCLE (PAPALIA ET AL., 2006)

**Figure 7:**
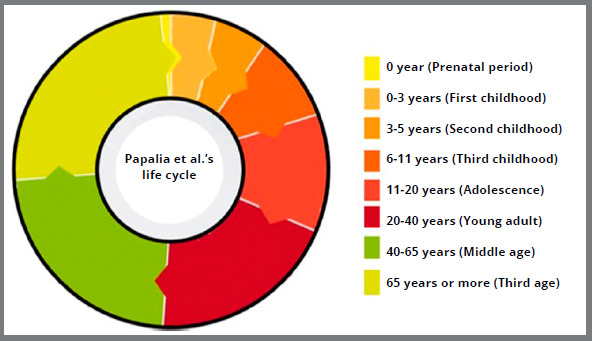
Graph with life cycle in 8 phases, based on the classification by Papalia et al.^16^ (2006).

Papalia et al.^16^ classified the life cycle into 8 phases besides age (Fig 7), considering physical, cognitive, and psychosocial development characteristics:

» Prenatal period: from conception to birth, when structure and basic body organs are formed.
» First childhood: from birth to 3 years. Corresponding to the phase in which all the senses work to different degrees, and great dependence on parents occurs.
» Second childhood: 3 to 6 years, when many cognitive skills are developed, the body has proportions more equivalent to that of an adult.
» Third childhood: 6 to 11 years, a period in which logical thinking is developed, and language and memory improve.
» Adolescence: 11 to approximately 20 years old, when a search for identity and maturity of the reproductive system occurs.
» Young adult: 20 to 40 years, being a period of the relative stability of personality traits, with an increased number of marital relationships and parenting.
» Middle age: 40 to 65 years, corresponding to the onset of some degree of loss of sensory capacity, health, and dexterity and, for women, hormonal changes because of menopause.
» Third age: 65 years and older, the last phase, being described as when retirement and a decrease in physical capacity and health commonly occur.

#### THE 8-PHASE LIFE CYCLE (ERIKSON, 2013)

**Figure 8:**
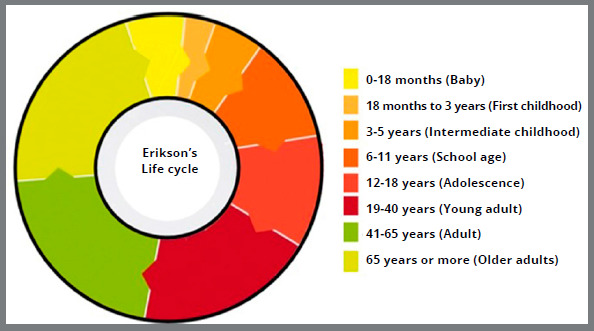
Graph with life cycle in 8 phases, based on the classification by Erik Erikson^17^ (2013).

For this classification (Fig 8), Erik Erikson^17^ addresses aspects related to emotional and interpersonal characteristics:

» Baby (0 to 18 months): oral-sensory phase, in which the person’s relationship is centered on the mother.
» First childhood (18 months to 3 years): phase characterized by the contradiction between impulses and social norms.
» Intermediate childhood (3 to 5 years): stage in which motor, language, and thinking skills fully develop.
» School-age (6 to 11 years): latency phase, in which school integration and formation of new interpersonal relationships occur.
» Adolescence (12 to 18 years): phase of acquisition of psychosocial identity.
» Young adult (19 to 40 years): a period in which the main point is the establishment of mature and lasting relationships.
» Adult (41 to 65 years): phase of personal affirmation at work and in the family.
» Older adults (65 years and beyond): last phase, in which the lived experiences are reassessed.

#### THE 8-PHASE LIFE CYCLE (BOGIN, 2020)

**Figure 9:**
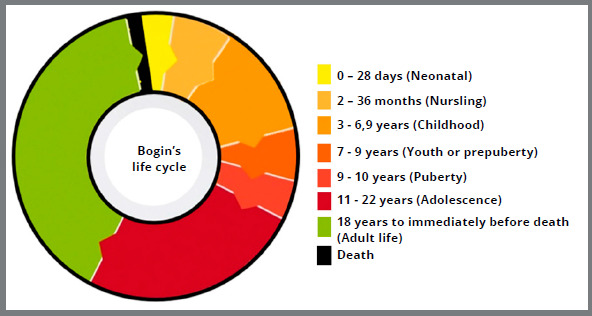
Graph with life cycle in 8 phases, based on the classification by Bogin^18^ (2020).

Barry Bogin^18^ classified the life cycle into 8 phases (Fig 9) that are described according to age group, length, biological, sociocultural, and cognitive signs:

» Neonatal stage (birth to 28 days): characterized by postnatal adaptation, ability to distinguish breast milk odor and reflexes guided by sound and light.
» Breastfed infant (from 2 months to the end of lactation, occurring around 30-36 months): with food coming totally or partially from milk, whether maternal or industrialized, associated with rapid cognitive, behavioral, and motor development.
  - *Early nursling* (2 to 12 months): is characterized by the introduction of other foods from 6 months onwards, the eruption of some deciduous teeth, and completion of the phase marked by bipedal walking.
  - *Late nursling* (12 to 30-36 months): development of verbal skills and finalized with the complete eruption of deciduous teeth, besides weaning.
» Childhood (3 to 6.9 years): period with moderate growth rate, well-developed bipedal walking, improvement in communication, independence, and self-care. This phase ends with the eruption of the first permanent molars and incisors and with adrenarche.
» Youth or prepuberty (7 to 9 years): characterized by a low growth rate, legal school age in most societies, and ability to feed independently.
» Puberty (9 to 10 years): neuroendocrine changes in the reproductive system, appearance of secondary sexual characteristics (darkening and increase in pubic/armpit hair, breast development in girls, and genital changes in boys), intensification of friendships and social activities.
» Adolescence (11 to 22 years): There is a growth spurt and intensification of social, economic, and sexual activities. The duration of this phase is different for girls (11 to 18 years) compared to boys (11 to 22 years).
  - *Prefertility* (11 to 13 years old): subcategory ending with menarche for girls and spermarche for boys.
  - *Fertility* (13 to 22 years): This phase is characterized by changes in lipid and muscle tissues related to sex, and increased physical and cognitive levels for work. This phase ends with the eruption of the third molars, the end of the fusion of epiphyses of long bones, and the maximum height reached.
» Adult life (from 18-22 years to immediately before death)
  - *Prime:* a phase that corresponds to the maximum performance of adult life, ending at the age of 35.
  - *Gradual decline* (35 to 50 years): It is observed the clinical detection of the first signs of physical degeneration, ending with menopause for women and, for men, reduced sperm quality.
  - *Transition or degenerative age* (50 years to senility): decreased cognitive and functional abilities.
  - *Senility:* variable duration phase, according to somatic and functional reserve levels.
  - *Death:* reduction of tissue and organ performance below the minimum support necessary for the maintenance of life.

#### THE 12-PHASE LIFE CYCLE (GONÇALVES, 2016)

**Figure 10:**
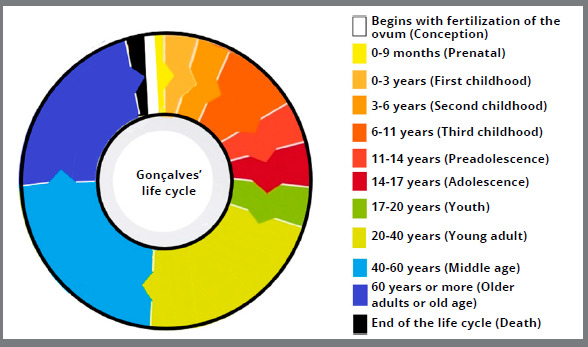
Graph with life cycle in 12 phases, based on Gonçalves^8^ classification (2016).

Gonçalves,^8^ based on a study by Papalia, Olds and Feldman,^19^ presented the 12 phases of the life cycle (Fig 10), with reference to age group, communication characteristics, development, interpersonal relationships, among other aspects evaluated in Psychology:

» Conception: begins with fertilization of the ovum.
» Prenatal (between 0 and 14 days up to 9 months): zygote (cell multiplication), embryo (beginning of organ formation) and fetus (body growth and improvement) stages.
» First childhood (0 to 3 years): in this phase the child develops two important skills: speech and locomotion.
» Second childhood (3 to 6 years): discovery of sexuality and construction of gender identity and frequent use of imagination.
» Third childhood (6 to 11 years old): slower physical development occurs, compared to the two previous stages, besides the beginning of school learning and friendships building.
» Preadolescence (11 to 14 years): intense body changes occur abruptly.
» Adolescence (14 to 17 years): is marked by the relationships building, in the form of friendship “groups”.
» Youth (17 to 20 years): transition to adult life, with increased social pressures and, therefore, with increased depression rates.
» Young adult (20 to 40 years): the physical and cognitive peak occurs, and is characterized by the themes of maternity/paternity, profession, and marriage.
» Middle age (40 to 60 years): beginning of decline in physical capacity, procreation, children leaving home, preparation for retirement.
» Older adults or old age (60 years and older): increased physical and cognitive frailty, retirement, and free time.
» Death: represents the end of the life cycle.

## DISCUSSION

The literature presents different classifications of the life cycle phases, which focus on chronology and social or biological issues. However, these classifications are not standardized, making communication between researchers and scholars difficult.

It was observed in the researched literature that among the classifications that were found, five described the life cycle in at least seven phases, concentrating more than half of the cycles before the adult phase. The phases related to old age were scarcely described. To classify the phases of the vital human cycle, besides biological age, morphological (measurements of height and weight compared to normative indices), skeletal (use of radiographs of the carpal bones of the hand and wrist to determine skeletal age), dental (sequence of eruption and tooth formation), and sexual aspects (analysis of primary and secondary sexual characteristics) should be considered. One could also include emotional (socialization) and mental aspects (cognitive/learning potential), self-perception (the person’s assessment of their ability), or perceptive age (personal assessment of development rate).^15^

The classifications by Gonçalves^8^ and Papalia et al.^*16*^ used chronological, biological, and behavioral criteria. Camarano’s 3-phase life cycle classification^1^ used criteria related to socio-cultural factors, showing that entering the labor market occurred during or after the person reached adulthood, without using the chronological time to define these phases. The classification proposed by Bogin and Smith^9^ in 5 phases presented factors beyond establishing age groups, such as developmental, sexual, and socio-cultural characteristics. The authors correlated dental development in the youth phase with the end of youth. They also related the youth and adolescence phases to the different ages of males and females. However, the authors did not mention how to evaluate the growth stage when citing the growth spurt, both to demarcate the beginning and the end of the adolescence phase. In adulthood, they describe the end of bone apposition and the beginning of resorption around the fifth decade of life.

Based on chronological age, the United Nations^14^ described the life cycle in six stages. The classification by Havighurst^13^ has 6 phases and is based on socio-cultural, learning, and function factors. However, it does not highlight the issue of the development of persons, which would be a use limitation for areas that involve growth and development. Gallahue et al.^*15*^ proposed a life cycle classification with well-established age groups, phases, and subdivisions, with chronological age as a criterion for definition.

A complete life cycle classification should contain comprehensive dental, growth, and aging elements and socio-cultural and behavioral characteristics. No classification was found that also included skeletal and dental development. The pubertal growth spurt can be assessed using carpal radiographs or vertebral maturation analysis (CS3 and CS4).^20^ The ascending curve of the pubertal growth spurt comprises the stages FD=, FP=, FM=, G1, Psi, R=, FDcap, S, and G2,^21^ ^,^ ^22^ described in Table 2 and shown in Figure 12. The peak of the pubertal growth spurt generally begins at 10 years and 7 months in females and at around 12 years and 7 months in males.^20^ ^,^ ^23^ This peak of pubertal growth spurt can be confirmed by the analysis of the carpal radiography of an individual between the phases FPcap (left-hand capping of the proximal phalanges of the middle finger) and FMcap (left-hand capping of the median phalanges of the middle finger). Dental aspects are also important markers of the life cycle, and the phase of dentition and its characteristics should be evaluated. The “Ugly Duckling Phase” in mixed dentition is a physiological stage in the development of human dentition that occurs between 8 and 10 years of age, and it is characterized by the distal inclination of the crowns and the presence of diastema between the upper incisors, overbite, and exaggerated overjet.^24^ This physiological example of a characteristic of dentition development exposes the importance of defining a more appropriate life cycle classification for the health area, especially for professionals who work with growth and development, because, at this age, at least eight nomenclatures could apply to this phase: youth,^9^ ^,^ ^14^ ^,^ ^18^ school age,^17^ adolescence,^9^ ^,^ ^13^ ^,^ ^15^ ^,^ ^17^ ^-^ ^19^ third childhood,^8^ ^,^ ^16^ childhood,^15^ second childhood,^13^ preadolescence,^8^ ^,^ ^13^ and puberty.^18^ In this way, the attempt to classify a research sample over the distinct phases of the life cycle so far proposed could generate bias, since several nomenclatures can be applied to the same phase, which is not always similar. This is the case of research that evaluates the presence of malocclusions and their relationship with the forms of breastfeeding and children’s habits, which would involve children between 2 and 4 years of age; these phases are classified in diverse ways: baby, first childhood, or middle childhood.^9^ ^,^ ^14^ ^,^ ^25^

**Figure 11:**
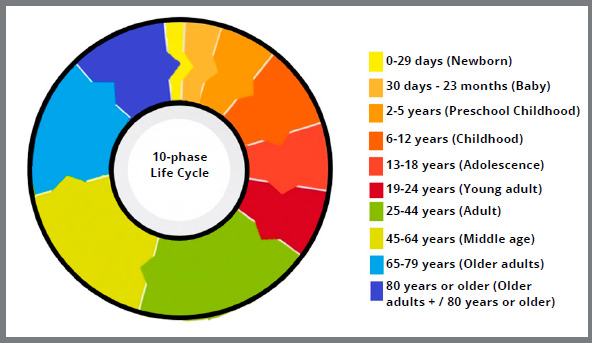
Graph with life cycle in 10 phases, based on the proposed classification.

Thus, in the present article, the authors suggest a classification for the life cycle, divided into 10 phases (Fig 11), based on dental, growth, physiological, socio-cultural, and behavioral aging characteristics. Chronological age must be considered. However, the classification should be based on the predominant characteristics in each phase, and not only on the age group.

### THE 10-PHASE LIFE CYCLE

#### 1. NEWBORN

» Breastfeeding.^26^
» Predental period, known as the period of gingival rollers, is characterized by the absence of teeth.^27^
» Physiological distocclusion: The mandible is retruded in relation to the maxilla, with a discrepancy of 3 to 5 mm.^28^
» Normally, from birth to 29 days.

#### 2. BABY

» Breastfeeding and, beginning at six months, solid food introduction.^18^
» Period of the deciduous dentition, which begins with the eruption of the first deciduous tooth.^2^
» Physiological growth of the mandible, enhanced by breastfeeding, promotes necessary muscular stimulation for development.^28^
» Great dependence on parents.^19^
» Normally, from 30 days to 23 months.

#### 3. PRESCHOOL CHILDHOOD

» The complete deciduous dentition marks the beginning of this life cycle’s phase.
» Development of cognitive skills.^9^
» The body assumes proportions like that of an adult.^16^
» Intersphenoidal synchondrosis fusion.^29^ ^,^ ^30^
» Normally, from 2 to 5 years old.

#### 4. CHILDHOOD

**Figure 12:**
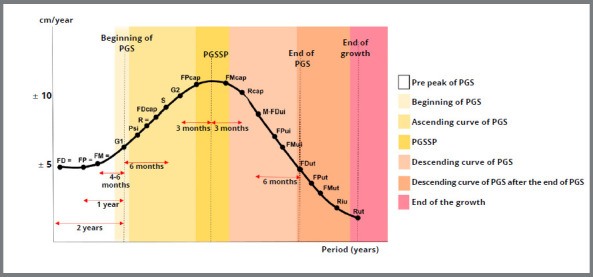
Phases of growth observed on the carpal radiograph illustrated in the growth curve (PGS = pubertal growth spurt, PGSSP = pubertal growth spurt speed peak) (Based on Mercadante^22^ 2001).

» Phase characterized by mixed dentition; starts with the eruption of the first permanent molars.^2^
» Ugly duckling phase.^24^
» Logical thinking development, language, and memory improvement.^16^
» Fusion of spheno-ethmoidal synchondrosis.^29^ ^,^ ^30^
» Usually, the individual is between the pre-peak phases of the pubertal growth spurt (between FD = and FM =), or in the beginning of the pubertal growth spurt (G1) or in the ascending phase of the pubertal growth spurt (Psi), or at the peak of the pubertal growth spurt (between FPcap and FMcap), determined by analyzing the middle finger of the left hand through carpal radiography^21,22^ (Table 1 and Fig 12).
» Normally, from 6 to 12 years old.

**Table 1:** Relationship between the phases observed on the carpal radiography and the growth characteristics (Based on Mercadante^22^ 2001).

Phase		Radiographic characteristics	Relationship with pubertal growth spurt (PGS)
Pre peak of PGS	FD =	Epiphyses of the distal phalanges with the same width as the diaphysis	About 2 years until the start of PGS
	FP =	Epiphyses of the proximal phalanges with the same width as the diaphysis	About 1 year until the start of PGS
	FM =	Epiphyses of the median phalanges with the same width as the diaphysis	About 4 to 6 months until the onset of PGS
Beginning of PGS	G1	Beginning of the appearance of the radiopaque hook of the hook bone	Marks the beginning of PGS
Ascending curve of PGS	Psi	Ossification of the pisiform bone	Occurs on the ascending curve of PSG; characterized by the appearance of secondary sexual characteristics
	R =	Epiphysis of the radius with the same width as the diaphysis	Occurs on the ascending curve of PGS
	FDcap	Capping of the distal phalanges	Occurs on the ascending curve of PGS
	S	Visualization of the sesamoid bone	Occurs on the ascending curve of the PGS; indicates that about 6 months have passed since the beginning of the PGS
	G2	Clear visualization of the hook of the hook bone	Occurs on the ascending curve of the PGS; indicates that there are about 3 months left for the PGS peak
Pubertal growth spurt speed peak (PGSSP)	FPcap	Capping of the proximal phalanges	Between FPcap and FMcap, it indicates that it is within the PGS (PGS velocity peak)
	FMcap	Capping of the median phalanges	Between FPcap and FMcap, it indicates that it is within the PGS (PGS velocity peak)
Descending curve of PGS	Rcap	Radius epiphysis capping	Occurs on the descending curve of the PGS; indicates that about 3 months have passed since the PGS peak velocity
	M-FDui	Menarche and beginning of union of distal phalanges	Occurs on the descending curve of the PGS; indicates that there are about 6 months left until the end of the PGS
	FPui	Beginning of the union of the proximal phalanges	Occurs on the descending curve of the PGS
	FMui	Beginning of the union of the median phalanges	Occurs on the descending curve of the PGS
Descending curve of PGS after the end of PGS	FDut	Total epiphyseal union of the distal phalanges	Marks the end of PGS, but not total growth
	FPut	Total epiphyseal union of the proximal phalanges	Occurs after the end of the PGS
	FMut	Total epiphyseal union of the median phalanges	Occurs after the end of the PGS
	Riu	Beginning of the epiphyseal union of the radius	Occurs after the end of the PGS
End of the growth	Rut	Complete epiphyseal union of the radius	Marks the end of maxillary growth; body, structural, and mandibular growth may extend for up to 1 or 2 years after total radius epiphyseal union (growth may occur as long as there is a cartilaginous radiolucent line between the epiphysis and diaphysis of the radius)

#### 5. ADOLESCENCE

» Transition from the late phase of the mixed dentition to permanent dentition: the eruption of permanent second molars and/or permanent canines.
» Post-menarche phase for girls (estimated growth of approximately five years more).^31^
» Vertebral maturation analysis: stages CS3 and CS4.^20^
» Through the analysis of the carpal radiograph, it can be determined whether the patient is at the ascending phase of the pubertal growth peak curve (between R= and G2), at the peak of the pubertal growth spurt (between FPcap and FMcap), at the beginning of the descending curve (Rcap and M-Fdui); at the descending curve of pubertal growth (between Fpui and Riu); or at the end of growth (Rut)^21^ ^,^ ^22^ (Table 1 and Fig 12).
» Search for identity and maturity of the reproductive system.^16^
» Normally, from 13 to 18 years.

#### 6. YOUNG ADULT

» Characterized by permanent dentures and the presence of third molars.
» Stability of personality traits.^16^
» Physical and cognitive peak.^8^ ^,^ ^19^
» Maximum performance of adult life.^18^
» Maturation of the cervical vertebrae shows that the patient is no longer growing;^20^ the carpal radiograph shows stages of the downward curve, or the end of the pubertal growth spurt (stages FPut, FMut, Riu or Rut),^21^ ^,^ ^22^ as shown in Table 1 and Figure 12.
» Beginning of synostosis of the coronal and sagittal sutures,^32^ and fusion of the spheno-occipital synchondrosis.^33^
» Normally, from 19 to 24 years.

#### 7. ADULT

» Complete permanent denture.
» Maturation of the cervical vertebrae and carpal radiography: the patient no longer shows growth (Rut stage).^20^ ^-^ ^22^
» Increase in the number of marital relationships and paternity.^16^
» Synostosis of the lambdoid suture.^32^
» Normally, from 25 to 44 years.

#### 8. MIDDLE AGE

» Characterized by mature permanent dentition, dental wear, arch length, and perimeter reduction, and decrease in the overbite, besides an increase in lower anterior crowding.^34^
» First signs of physical degeneration, characterized by menopause for women,^35^ and reduced sperm quality for men.^18^
» Affected reproductive capacity.^8^
» Normally, from 45 to 64 years old.

#### 9. OLDER ADULTS

» Greater susceptibility to tooth fractures and loss; use of dental prostheses and/or implants.
» Teeth in more yellowish, brown, or gray tones; teeth with greater susceptibility to erosion, abrasion, plaque accumulation, and periodontal disease.^36^
» Adaptation of the stomatognathic system in the face of morpho-functional conditions, which become slower, uncoordinated, and adapted to the structural damage that occurs over the years.^37^
» Process of bone resorption in the maxilla and mandible, increasing its porosity; jawbone thickness reduction and mandibular angle decrease.^36^
» Decreased proximal femur mineral density, increasing the risk of hip fractures.^38^
» Increased physical and cognitive frailty.^8^
» In general, from 65 to 79 years old.

#### 10. OLDER ADULTS + (80 YEARS OR OLDER)

» Partial or total absence of teeth associated with the use of prostheses.
» Partial or complete obliteration of the volume of the canals and pulp chamber.^39^
» Reduced dry mouth perception and difficulty for eating; the higher need for prosthesis.^36^ ^,^ ^37^
» Higher frequency of chronic pain.^40^

The distinct phases of the life cycle proposed in this study can be seen in Table 2, distributed according to the characteristics presented.

**Table 2:** Phases of the proposed life cycle (The 10-phase Life Cycle), with the striking and frequent characteristics of each phase.

Phase (chronological age)		Dentition	Dental features	Skeletal and developmental features	Growth stages (carpal radiograph)	General features
Pre peak of pubertal growth spurt (PGS)	Newborn (From birth to 29 days)	Period of gingival rollers	Absence of teeth	Physiological dystocclusion	Not applicable	Complete dependence on parents; Exclusive breastfeeding
	Baby (From 30 days to 23 months)	Deciduous	Beginning of eruption of deciduous teeth	Reduction of physiological distoclusion	Not applicable	Great dependence on parents; introduction of solid foods; speech evolution
	Preschool Childhood (2 -5 years)	Deciduous/early mixed	Complete deciduous dentition. Determination of the occlusion key of deciduous second molars Transition to mixed dentition, with eruption of first molars and permanent incisors.	Intersphenoidal synchondrosis fusion	Generally does not apply	Many cognitive skills develop
Beginning of PGS, or Ascending curve of PGS, or Pubertal growth spurt speed peak (PGSSP)	Childhood (6 - 12 years)	Mixed	Ugly duckling phase Eruption of permanent canines and premolars	Spheno-ethmoidal synchondrosis fusion	Ascending curve of pubertal growth spurt Proximity to the peak of the pubertal growth spurt	Development of logical thinking, improvement of language and memory; Appearance of secondary sexual characters; Period of menarche (girls)
Ascending curve of PGS, or Pubertal growth spurt speed peak (PGSSP), or Descending curve of PGS	Adolescence (13 - 18 years)	Mixed late	Eruption of permanent canines	Presence of growth (stages CS3 to CS5 of cervical vertebrae maturation)	Peak of the pubertal growth spurt	Appearance of secondary sexual characters Period of menarche/post-menarche (girls);
		Permanent	Presence of permanent second molars; Possible eruption of third molars		Descending curve of pubertal growth	Search for the identity and maturity of the reproductive system
Descending curve of PGS After the end of PGS, or End of the growth	Young adult (19 - 24 years)	Permanent	Presence of third molars	Final or absence of growth (stages CS5 to CS6 of cervical vertebrae maturation)	Descending curve	Stability of personality traits; Physical and cognitive peak
				Beginning of synostosis of the coronal and sagittal sutures, and fusion of the spheno-occipital synchondrosis	Or the end of the pubertal growth spurt	Maximum performance of adult life
End of the growth	Adult (25 - 44 years)	Permanent	Complete permanent dentition	Synostosis of the lambdoid suture	No longer shows growth	Increase in the number of marital relationships; paternity/maternity
	Middle age (45- 64 years)	Permanent	Possible tooth loss due to fractures, trauma; Dental wear; Reduction in the length and perimeter of the arch; increased overbite; and lower anterior crowding.	Fused sutures and synchondroses	No longer shows growth	First signs of physical degeneration; Decreased sperm quality Menopause / Andropause
	Older adults (65 - 79 years)	Permanent	Eventual partial or total dental absences and use of dental prostheses; Teeth in more yellowish, brown, or gray tones; Erosion, abrasion.	Fused sutures and synchondroses; Greater resorption in the maxilla and mandible.	No longer shows growth	Increased physical and cognitive fragility; higher risk of bone fractures. Reduced muscle tone
	Older adults + (80 years or older)	Permanent	Eventual partial or total dental absences and use of dental prostheses; Partial or complete obliteration of the volume of the canals and pulp chamber; reduced salivary volume; difficulty eating.	Fused sutures and synchondroses	No longer shows growth	Frequent chronic pain Reduced muscle tone

This article was written in response to the need for a life cycle classification that provides clear and well-defined boundaries for its phases, specifically for use in scientific research within the healthcare field. Moreover, due to the growing life expectancy, some existing classifications grouped individuals into vastly different stages of maturation, cognition, and requirements. As a result, the proposed life cycle classification seeks to expand the viewpoint and promote contemplation on this matter.

## CONCLUSION

In this study, we conducted a comprehensive review of existing studies on life cycle classifications, focusing on their distinct phases. Based on the present analysis, the authors propose a classification that considers dental, growth, physiological aging, sociocultural, and behavioral characteristics. The primary aim of this classification is to enhance communication among healthcare professionals, particularly those involved in human growth, development, and aging. By providing a framework that encompasses these various aspects, the authors believe it will facilitate a more comprehensive understanding and effective collaboration in this field.
